# Supplementary material for: First-in-Human, Randomized, Placebo-Controlled, Double-Blind Phase 1 Study to Assess in Healthy Adults the Safety and Immunogenicity of Intramuscularly Administered AAVLP-HPV Vaccine
Source: medRxiv. 2026 Aug 10:2026.08.07.26359762. Preprint. [Version 1] doi: 10.64898/2026.08.07.26359762 (PMC13640826; doi:10.64898/2026.08.07.26359762)
Supplement: Supplementary file 1 [file media-1.pdf]

Supplementary Table S1: Serum antibody titer determined by HPV L2 peptide ELISA. **Geometric mean titer** [95% Confidence Interval] in Active (**A**) and placebo (**P**) arms.

**Day**	**HPV16 L2**		**HPV31 L2**		**HPV5 L2**		**HPV15 L2**		**HPV20 L2**		**HPV38 L2**		**HPV76 L2**	
	**A**	**P**	**A**	**P**	**A**	**P**	**A**	**P**	**A**	**P**	**A**	**P**	**A**	**P**
-1	**407**<br>286-579	**623**<br>306-1268	**493**<br>349-696	**618**<br>310-1231	**605**<br>437-838	**771**<br>295-2014	**1523**<br>1069-2171	**2623**<br>1513-4548	**897**<br>576-1395	**1611**<br>893-2906	**2926**<br>2133-4013	**3867**<br>1829-8177	**878**<br>432-1782	**898**<br>359-2249
15	**1508**<br>960-2368	**457**<br>296-704	**1108**<br>734-1672	**432**<br>289-646										
57	**3268**<br>1791-5962	**867**<br>460-1636	**2886**<br>1448-5751	**634**<br>251-1602										
71	**6058**<br>2976-12330	**733**<br>480-1120	**5373**<br>2378-12139	**606**<br>331-1111	**3440**<br>1365-8672	**691**<br>300-1594	**7060**<br>2880-17303	**2035**<br>1718-2412	**5700**<br>2177-14925	**1232**<br>938-1618	**6604**<br>3731-11691	**2746**<br>1493-5048	**3762**<br>1490-9498	**769**<br>340-1740
181	**1640**<br>730-3687	**645**<br>465-894	**1634**<br>750-3560	**614**<br>398-949										
194	**4526**<br>2162-9475	**541**<br>332-882	**4695**<br>2069-10655	**527**<br>335-827	**3888**<br>1937-7803	**778**<br>352-1720	**7785**<br>3873-15649	**1933**<br>1634-2286	**5421**<br>2592-11337	**1188**<br>929-1520	**6441**<br>3583-11579	**2799**<br>1393-5624	**4039**<br>1866-8742	**803**<br>406-1589
240	**3358**<br>1509-7471	**666**<br>350-1268	**3301**<br>1370-7955	**634**<br>287-1400										
365	**1671**<br>828-3374	**524**<br>302-909	**1591**<br>738-3429	**544**<br>475-622	**1488**<br>808-2740	**810**<br>348-1887	**3044**<br>1646-5630	**2017**<br>1323-3075	**2021**<br>1005-4064	**1289**<br>809-2056	**3816**<br>2315-6291	**2800**<br>1250-6272	**1919**<br>884-4166	**840**<br>482-1462

Supplementary Table S2: Proportion of responders with 2-, 5-, and 10-fold increases in serum IgG titer against L2 peptide by HPV genotype.

Genotype	Day	Placebo		Active			
		n	x2	n	x2	x5	x10
HPV16	71	4	0.0%	15	100.0%***	73.3%*	60.0%
	194	4	0.0%	14	92.9%**	71.4%*	50.0%
	365	4	0.0%	14	85.7%**	21.4%	14.3%
HPV31	71	4	0.0%	15	86.7%**	66.7%*	53.3%
	194	4	0.0%	14	85.7%**	64.3%	57.1%
	365	4	0.0%	14	64.3%	42.9%	14.3%
HPV5	71	4	0.0%	15	66.7%*	53.3%	33.3%
	194	4	0.0%	14	78.6%*	50.0%	35.7%
	365	4	0.0%	14	42.9%	21.4%	7.1%
HPV15	71	4	0.0%	15	66.7%*	40.0%	26.7%
	194	4	0.0%	14	92.9%**	71.4%	50.0%
	365	4	0.0%	14	35.7%	7.1%	7.1%
HPV20	71	4	0.0%	15	73.3%*	46.7%	46.7%
	194	4	0.0%	14	85.7%**	64.3%	21.4%
	365	4	0.0%	14	64.3%	14.3%	7.1%
HPV38	71	4	0.0%	15	53.3%	20.0%	13.3%
	194	4	0.0%	14	42.9%	21.4%	7.1%
	365	4	0.0%	14	14.3%	7.1%	7.1%
HPV76	71	4	0.0%	15	66.7%*	33.3%	20.0%
	194	4	0.0%	14	64.3%	42.9%	28.6%
	365	4	0.0%	14	42.9%	14.3%	7.1%

Supplementary Figure 1. Geometric Mean (95% CI) Anti HPV2 Neutralizing Antibody Titer Versus Time Profiles Following 3 Intramuscular Injections of AAVLP-HPV Vaccine 20 µg or Placebo Vaccine on Days 1, 57, and 180.

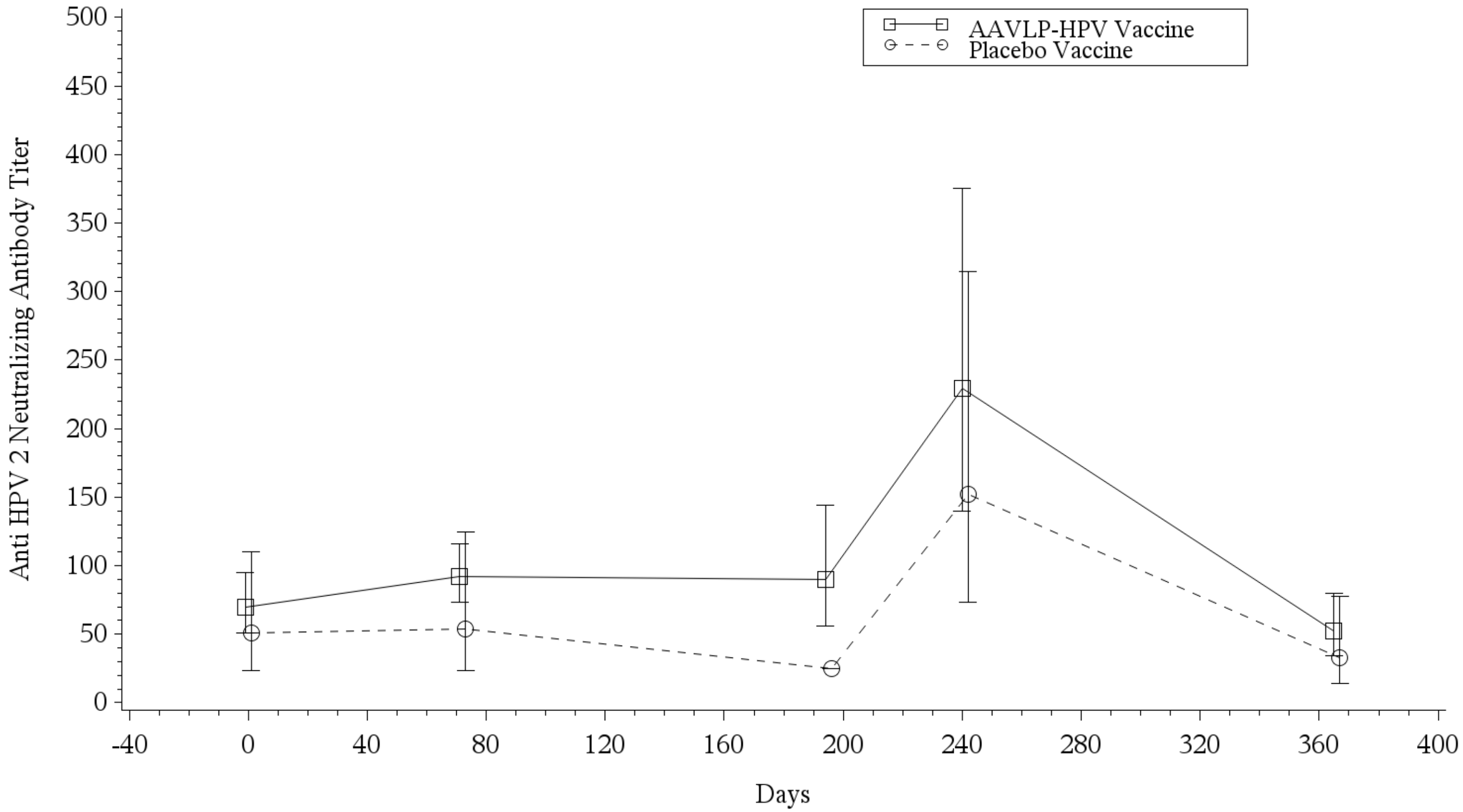

Placebo Vaccine is shifted to the right for ease of reading.
Program: /CA21491/sas_prg/pksas/ada/meangraph.sas 12SEP2022 8:03

Supplementary Figure 2. Geometric Mean (95% CI) Anti HPV3 Neutralizing Antibody Titer Versus Time Profiles Following 3 Intramuscular Injections of AAVLP-HPV Vaccine 20 µg or Placebo Vaccine on Days 1, 57, and 180.

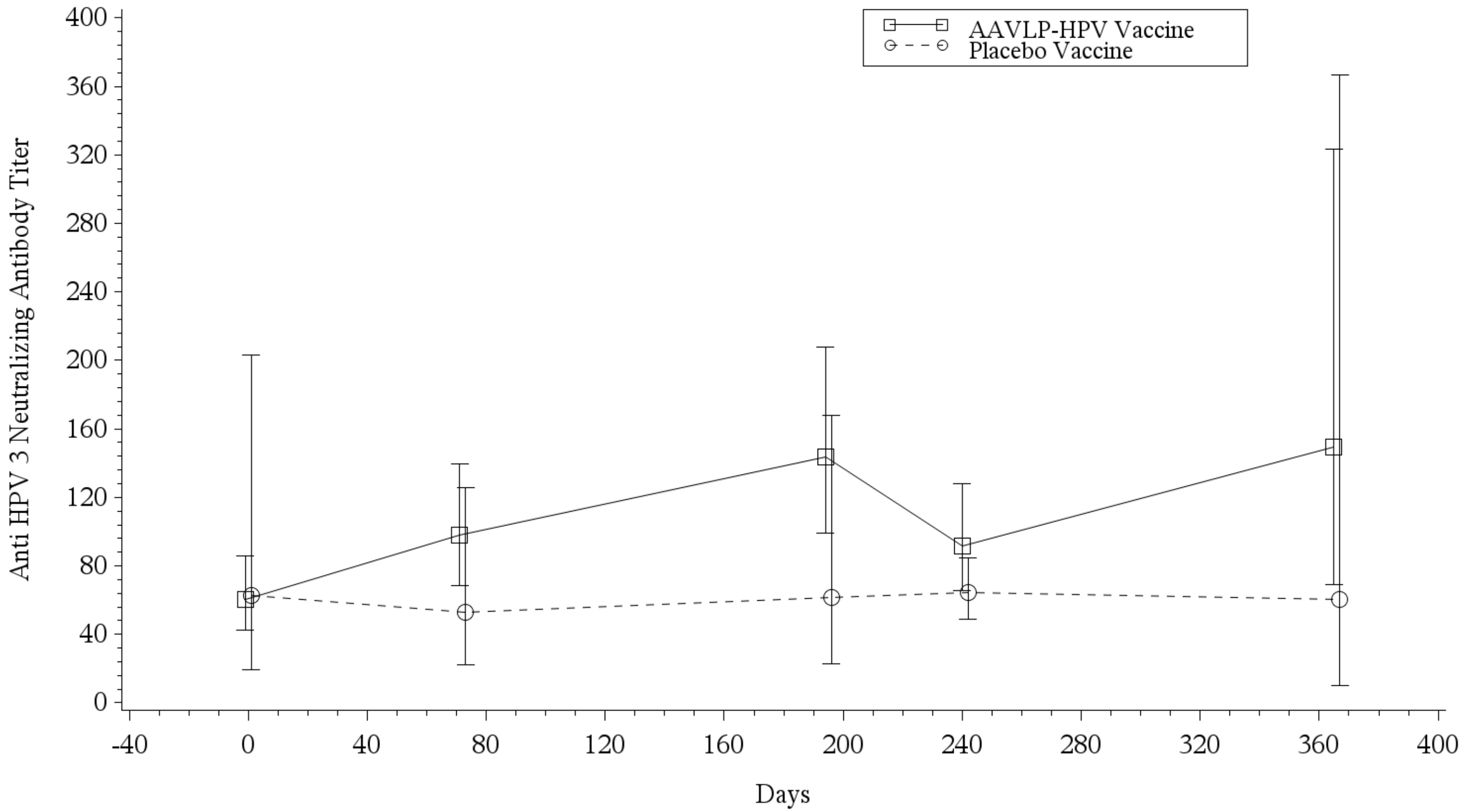

Placebo Vaccine is shifted to the right for ease of reading.
Program: /CA21491/sas_prg/pksas/ada/meangraph.sas 12SEP2022 8:03

Supplementary Figure 3. Geometric Mean (95% CI) Anti HPV6 Neutralizing Antibody Titer Versus Time Profiles Following 3 Intramuscular Injections of AAVLP-HPV Vaccine 20 µg or Placebo Vaccine on Days 1, 57, and 180.

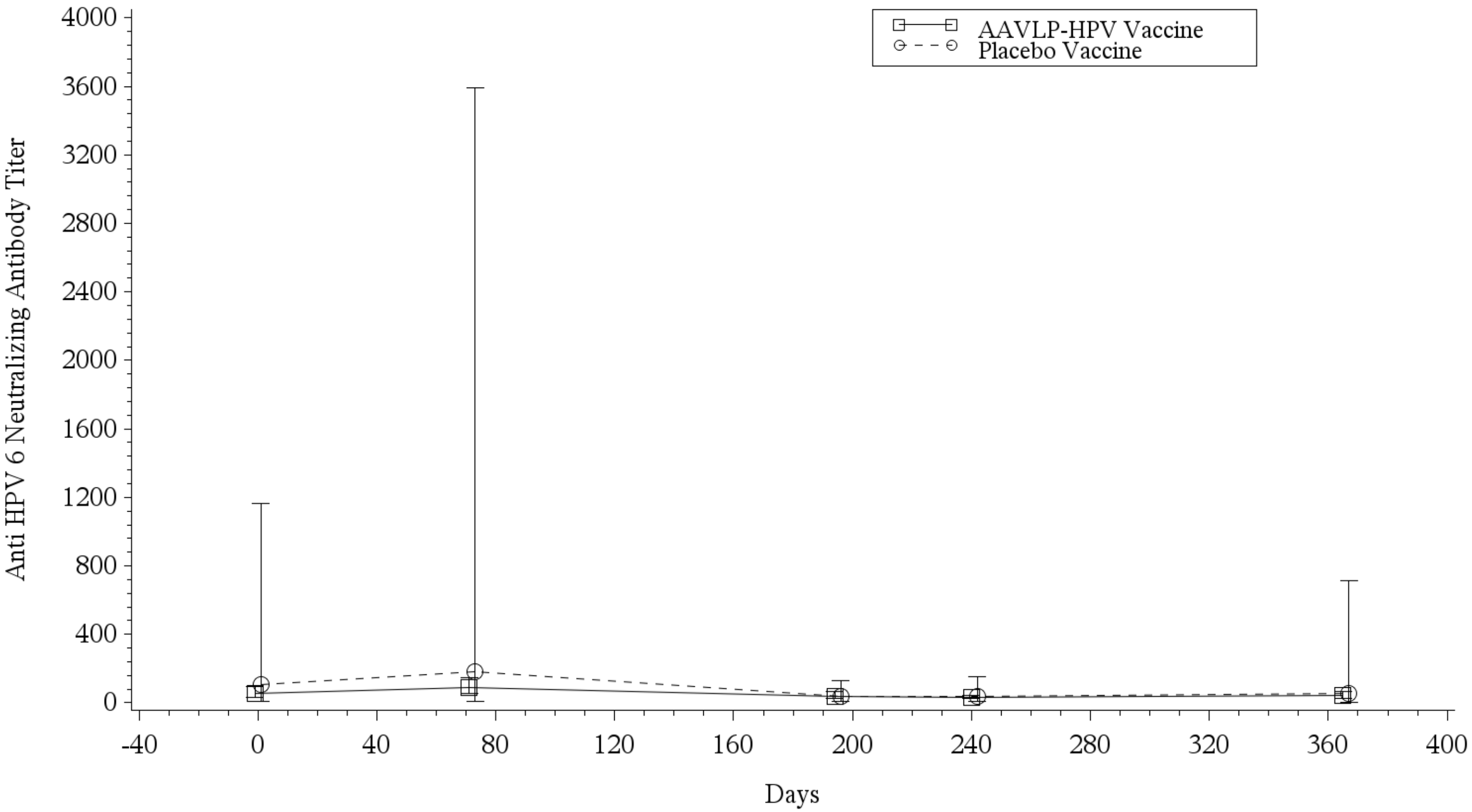

Placebo Vaccine is shifted to the right for ease of reading.
Program: /CA21491/sas_prg/pksas/ada/meangraph.sas 12SEP2022 8:03

Supplementary Figure 4. Geometric Mean (95% CI) Anti HPV11 Neutralizing Antibody Titer Versus Time Profiles Following 3 Intramuscular Injections of AAVLP-HPV Vaccine 20 µg or Placebo Vaccine on Days 1, 57, and 180.

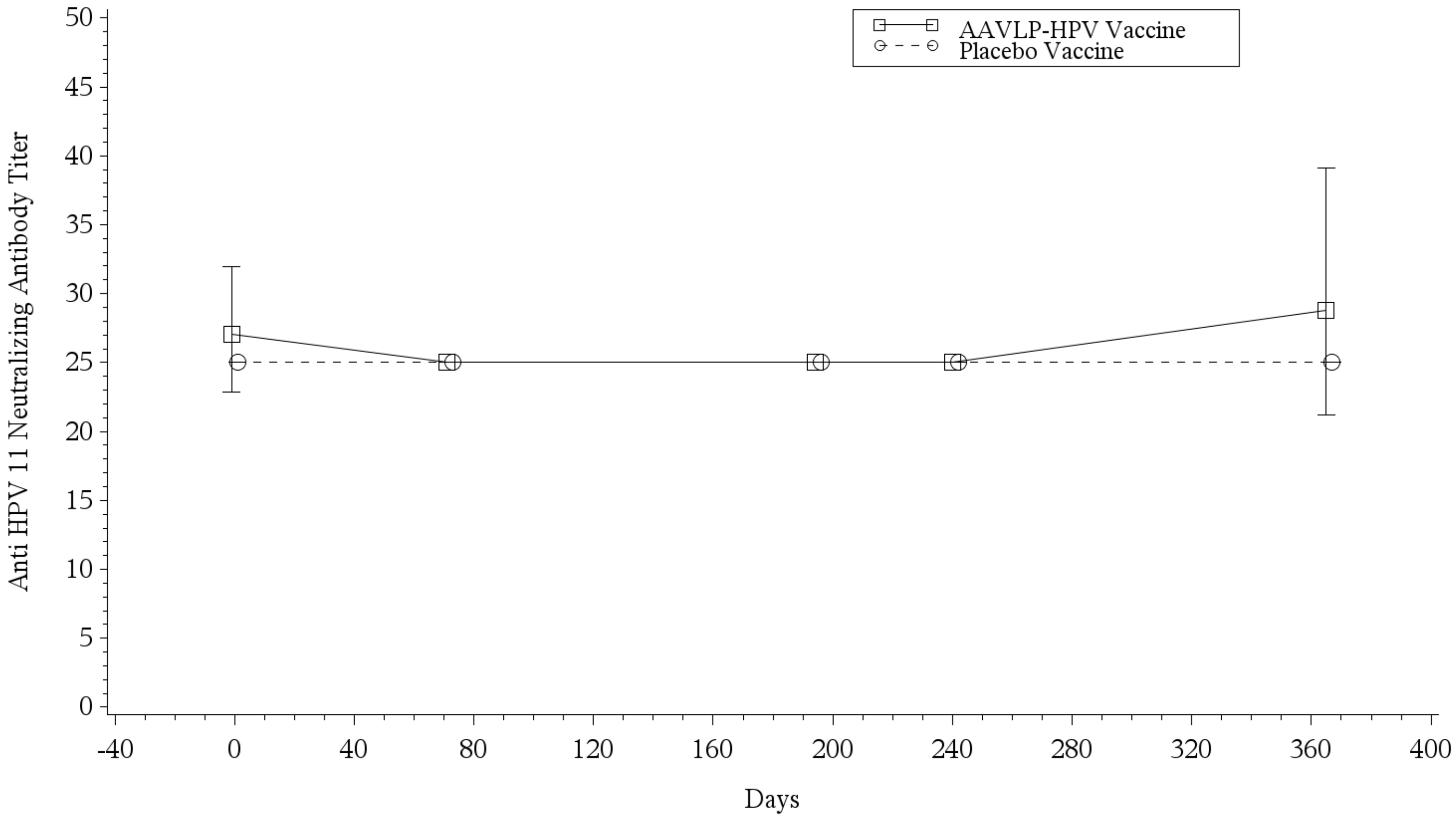

Placebo Vaccine is shifted to the right for ease of reading.
Program: /CA21491/sas_prg/pksas/ada/meangraph.sas 12SEP2022 8:03

Supplementary Figure 5. Geometric Mean (95% CI) Anti HPV18 Neutralizing Antibody Titer Versus Time Profiles Following 3 Intramuscular Injections of AAVLP-HPV Vaccine 20 µg or Placebo Vaccine on Days 1, 57, and 180.

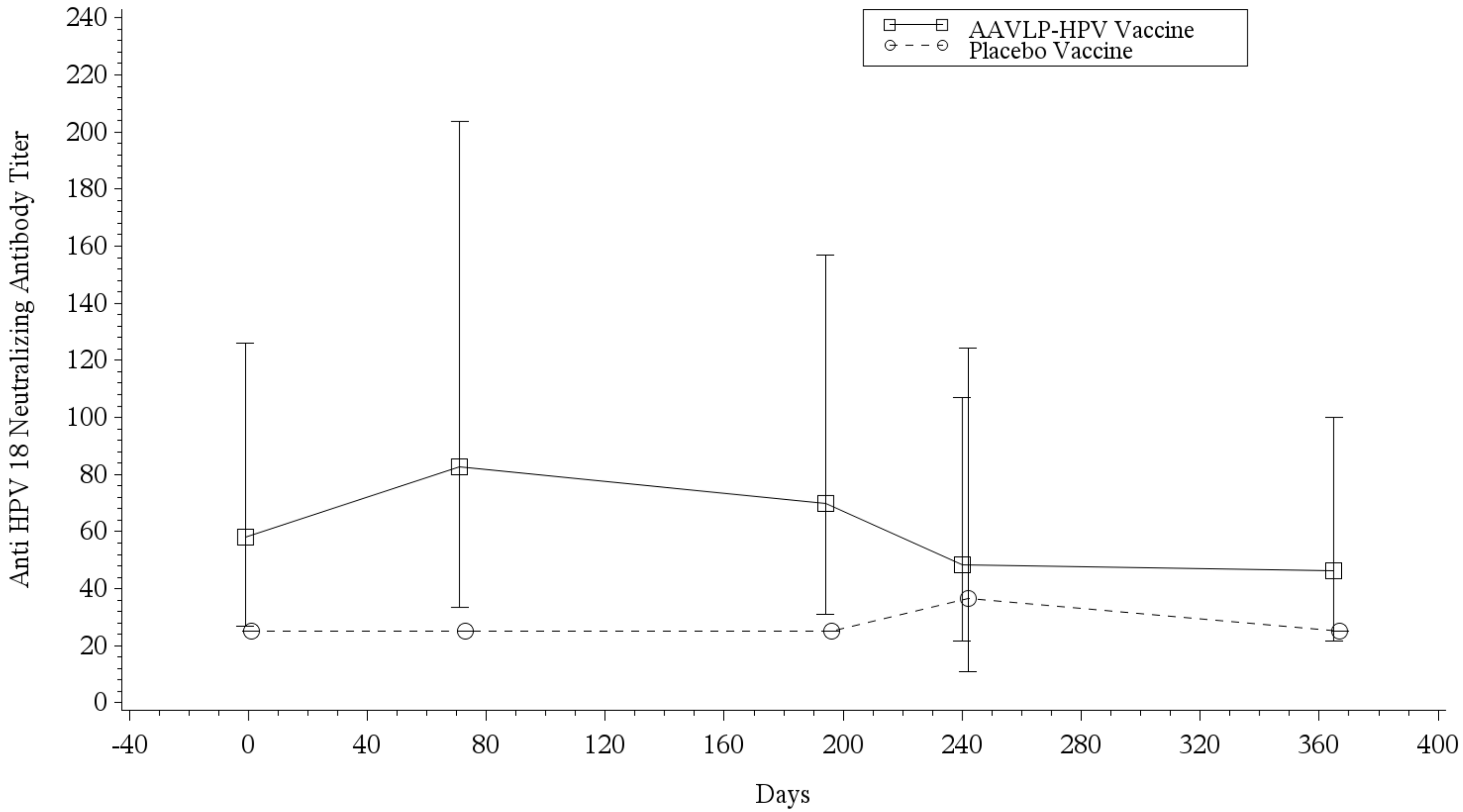

Placebo Vaccine is shifted to the right for ease of reading.
Program: /CA21491/sas_prg/pksas/ada/meangraph.sas 12SEP2022 8:03

Supplementary Figure 6. Geometric Mean (95% CI) Anti HPV33 Neutralizing Antibody Titer Versus Time Profiles Following 3 Intramuscular Injections of AAVLP-HPV Vaccine 20 μg or Placebo Vaccine on Days 1, 57, and 180.

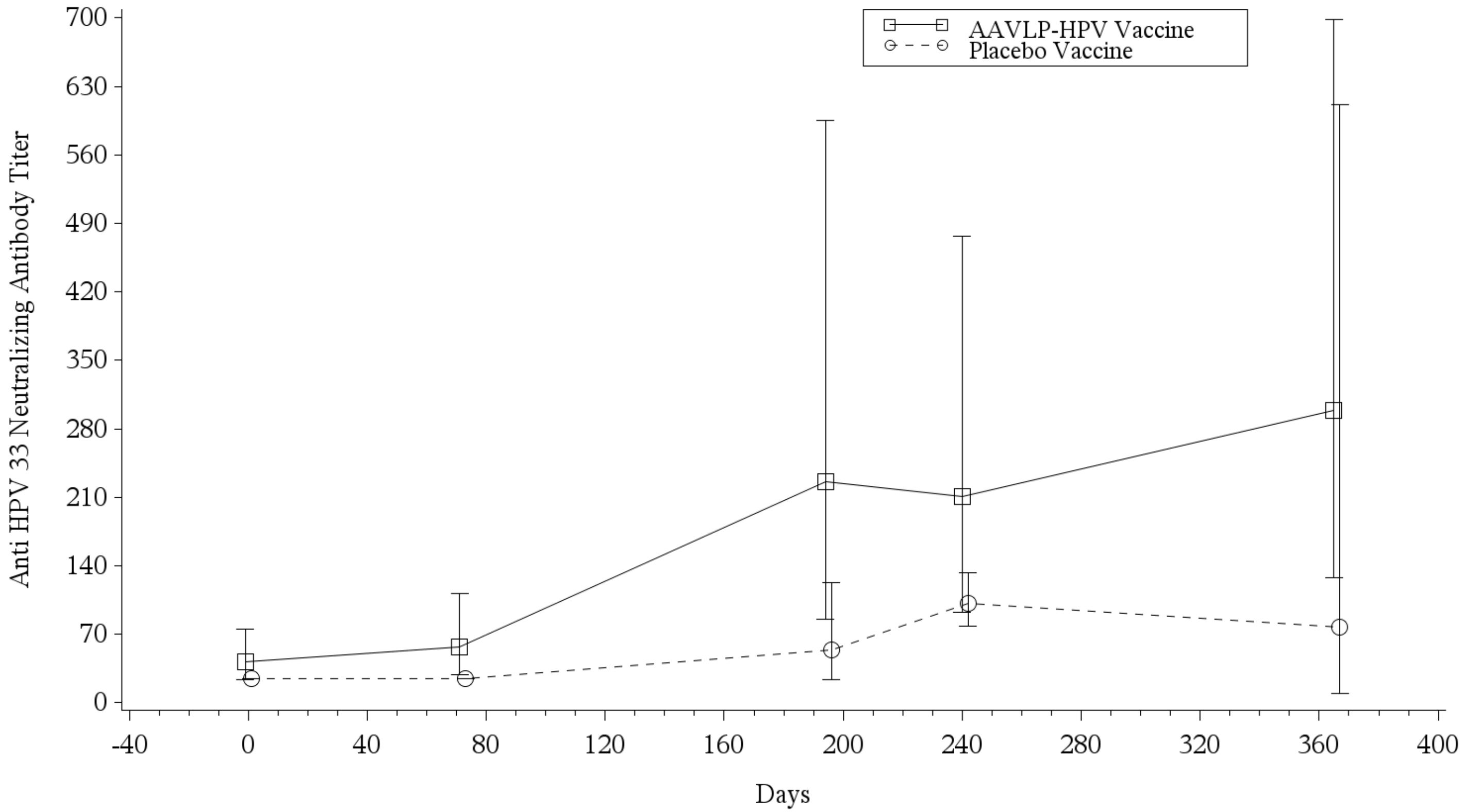

Placebo Vaccine is shifted to the right for ease of reading.
Program: /CA21491/sas_prg/pksas/ada/meangraph.sas 12SEP2022 8:04

Supplementary Figure 7. Geometric Mean (95% CI) Anti HPV35 Neutralizing Antibody Titer Versus Time Profiles Following 3 Intramuscular Injections of AAVLP-HPV Vaccine 20 µg or Placebo Vaccine on Days 1, 57, and 180.

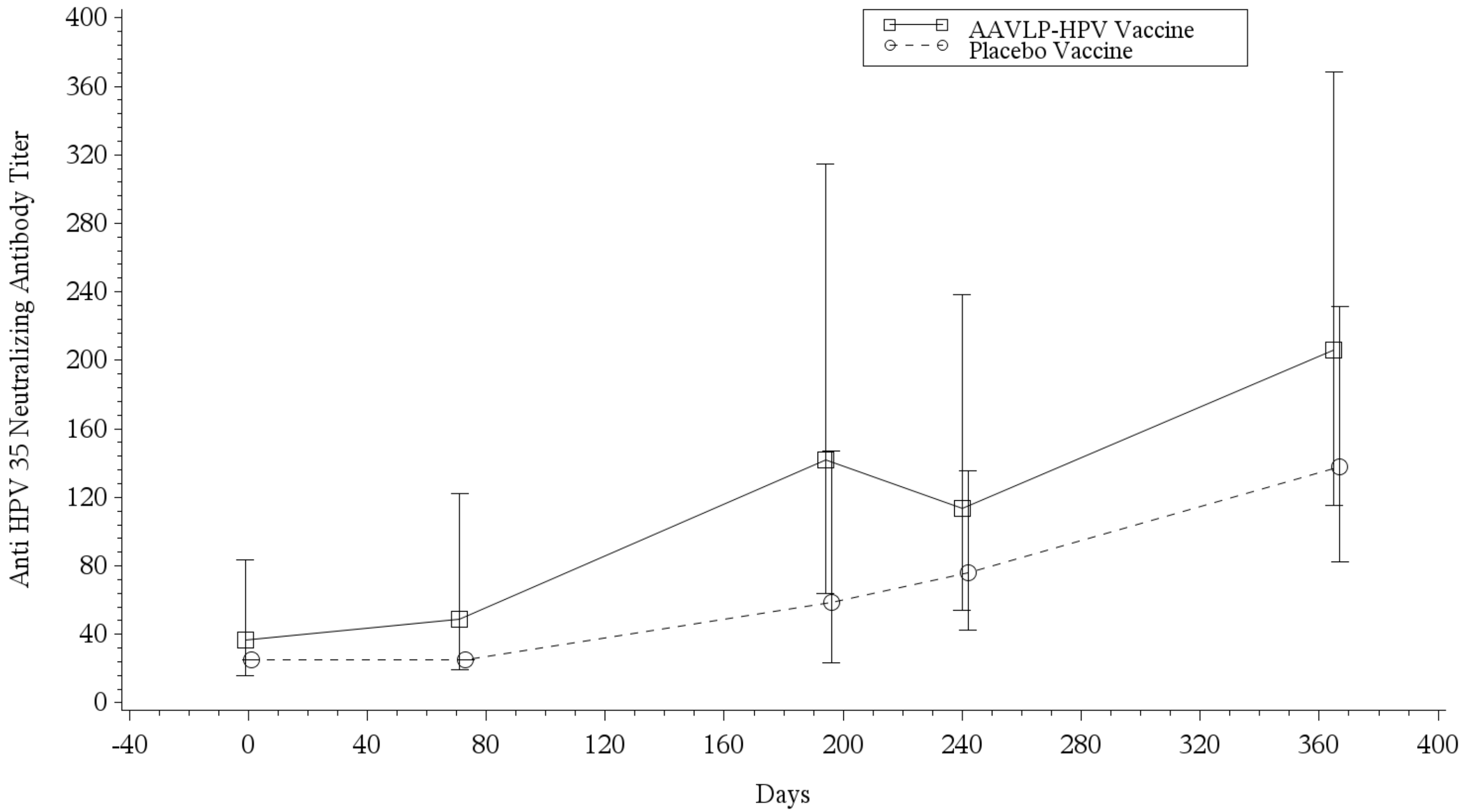

Placebo Vaccine is shifted to the right for ease of reading.
Program: /CA21491/sas_prg/pksas/ada/meangraph.sas 12SEP2022 8:04

Supplementary Figure 8. Geometric Mean (95% CI) Anti HPV38 Neutralizing Antibody Titer Versus Time Profiles Following 3 Intramuscular Injections of AAVLP-HPV Vaccine 20 µg or Placebo Vaccine on Days 1, 57, and 180.

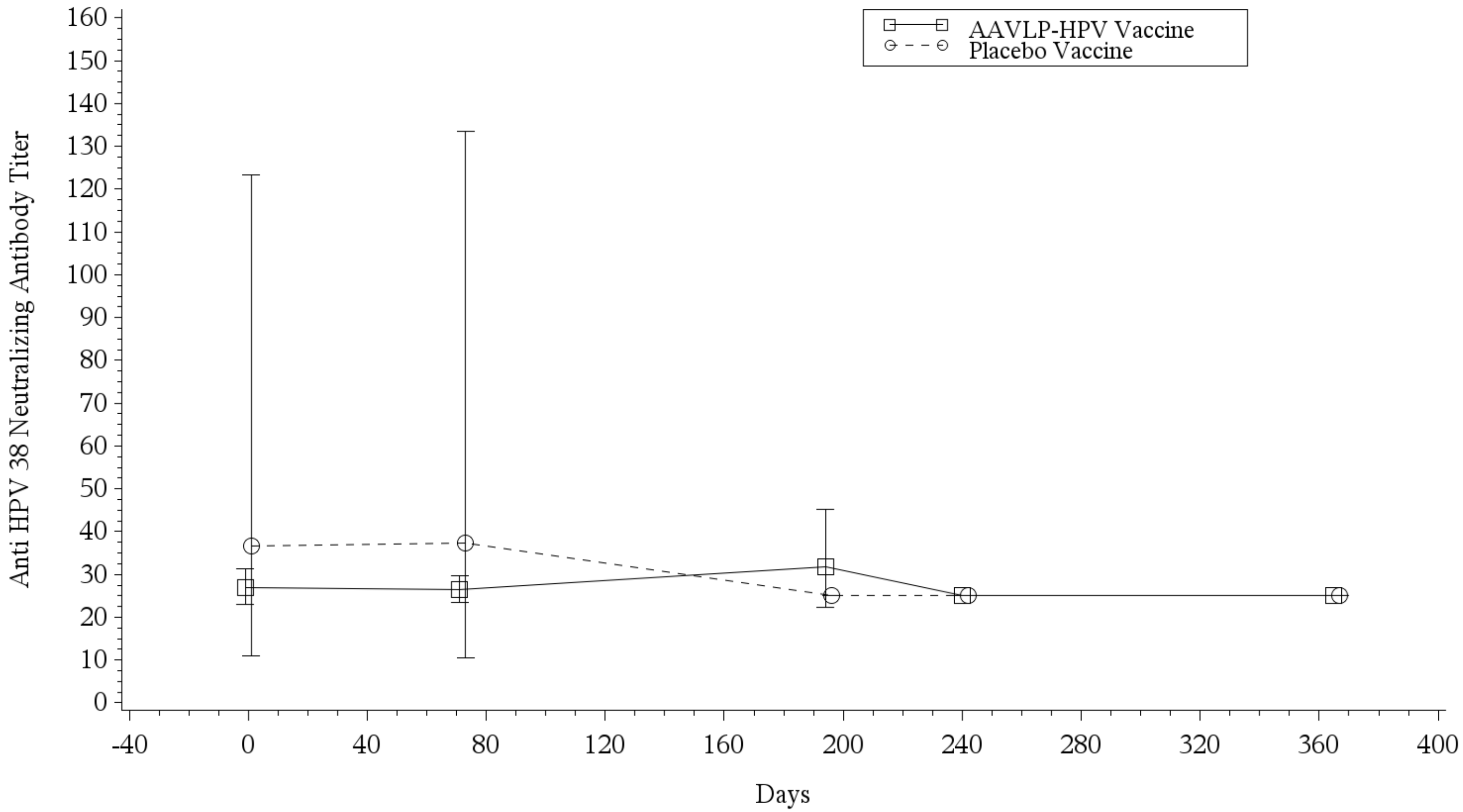

Placebo Vaccine is shifted to the right for ease of reading.
Program: /CA21491/sas_prg/pksas/ada/meangraph.sas 12SEP2022 8:04

Supplementary Figure 9. Geometric Mean (95% CI) Anti HPV39 Neutralizing Antibody Titer Versus Time Profiles Following 3 Intramuscular Injections of AAVLP-HPV Vaccine 20 µg or Placebo Vaccine on Days 1, 57, and 180.

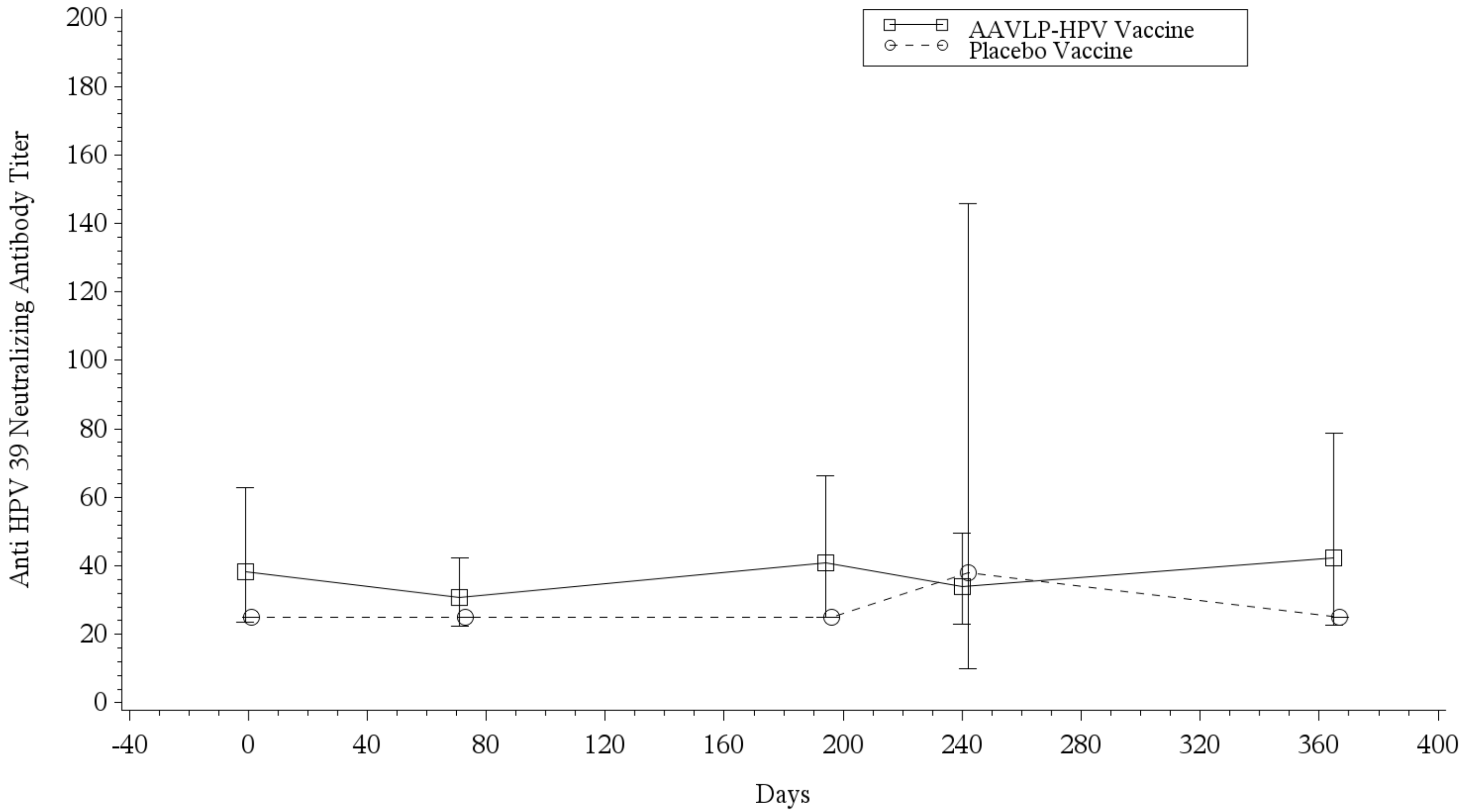

Placebo Vaccine is shifted to the right for ease of reading.
Program: /CA21491/sas_prg/pksas/ada/meangraph.sas 12SEP2022 8:04

Supplementary Figure 10. Geometric Mean (95% CI) Anti HPV45 Neutralizing Antibody Titer Versus Time Profiles Following 3 Intramuscular Injections of AAVLP-HPV Vaccine 20 µg or Placebo Vaccine on Days 1, 57, and 180.

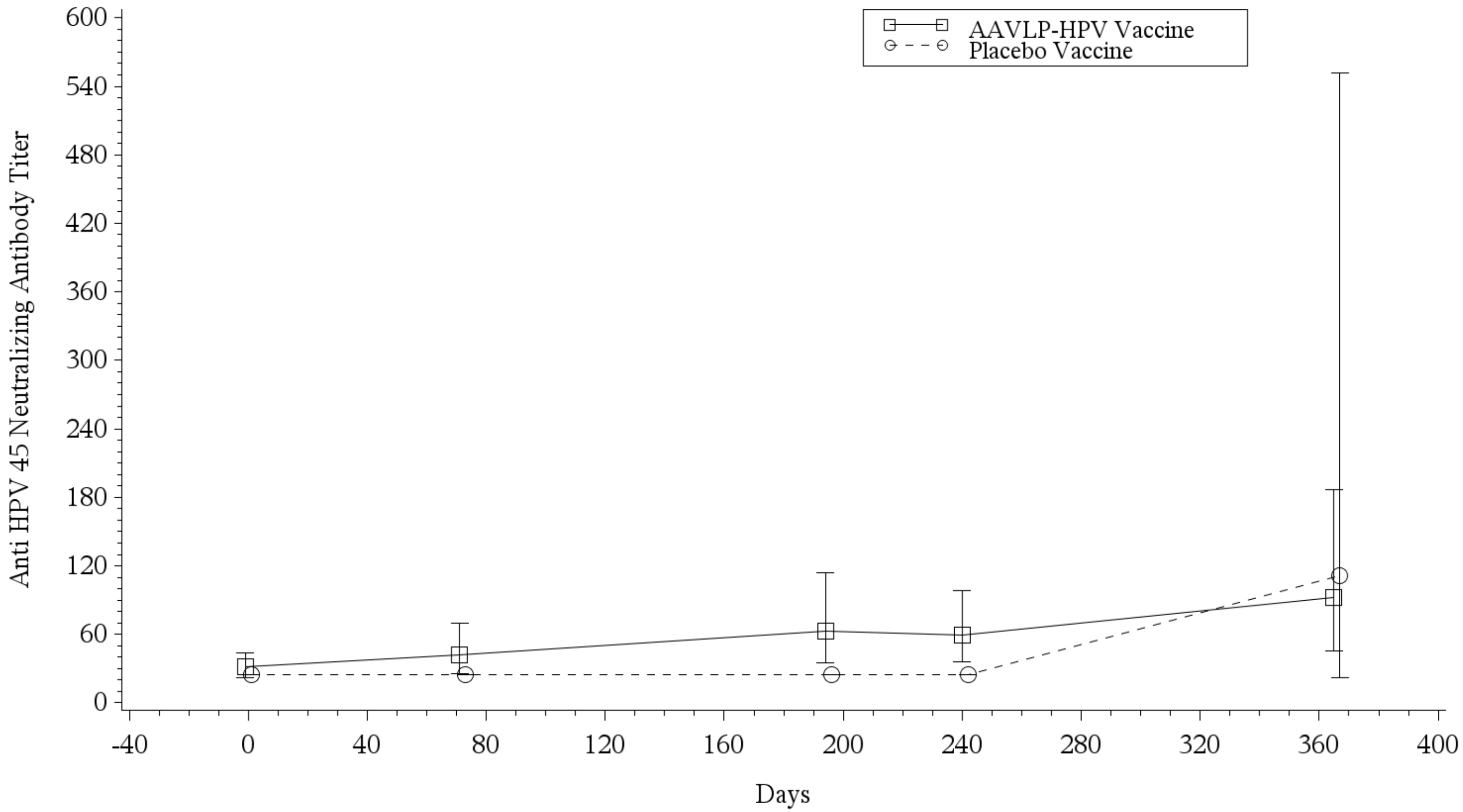

Placebo Vaccine is shifted to the right for ease of reading.
Program: /CA21491/sas_prg/pksas/ada/meangraph.sas 12SEP2022 8:04

Supplementary Figure 11. Geometric Mean (95% CI) Anti HPV51 Neutralizing Antibody Titer Versus Time Profiles Following 3 Intramuscular Injections of AAVLP-HPV Vaccine 20 µg or Placebo Vaccine on Days 1, 57, and 180.

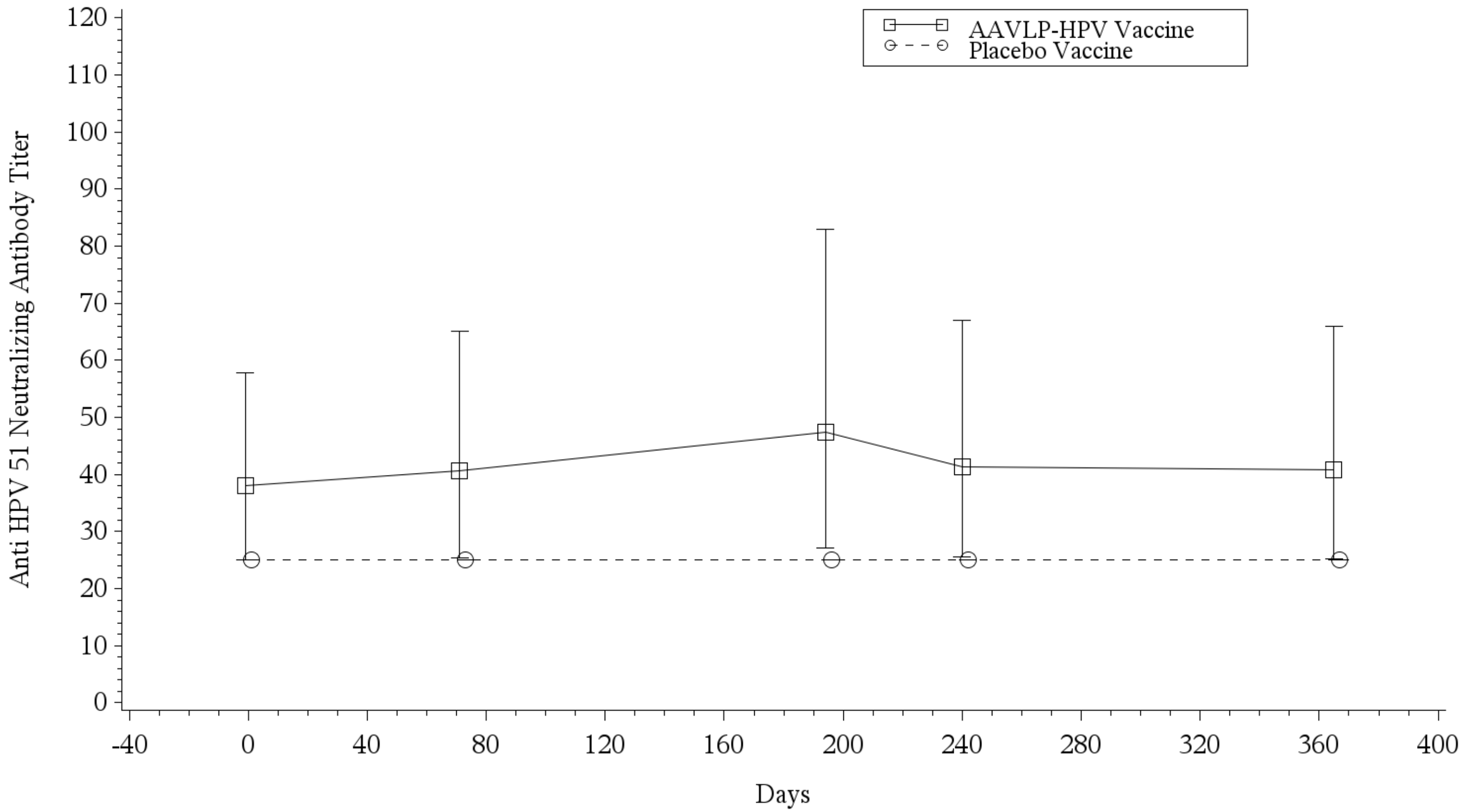

Placebo Vaccine is shifted to the right for ease of reading.
Program: /CA21491/sas_prg/pksas/ada/meangraph.sas 12SEP2022 8:04

Supplementary Figure 12. Geometric Mean (95% CI) Anti HPV52 Neutralizing Antibody Titer Versus Time Profiles Following 3 Intramuscular Injections of AAVLP-HPV Vaccine 20 μg or Placebo Vaccine on Days 1, 57, and 180.

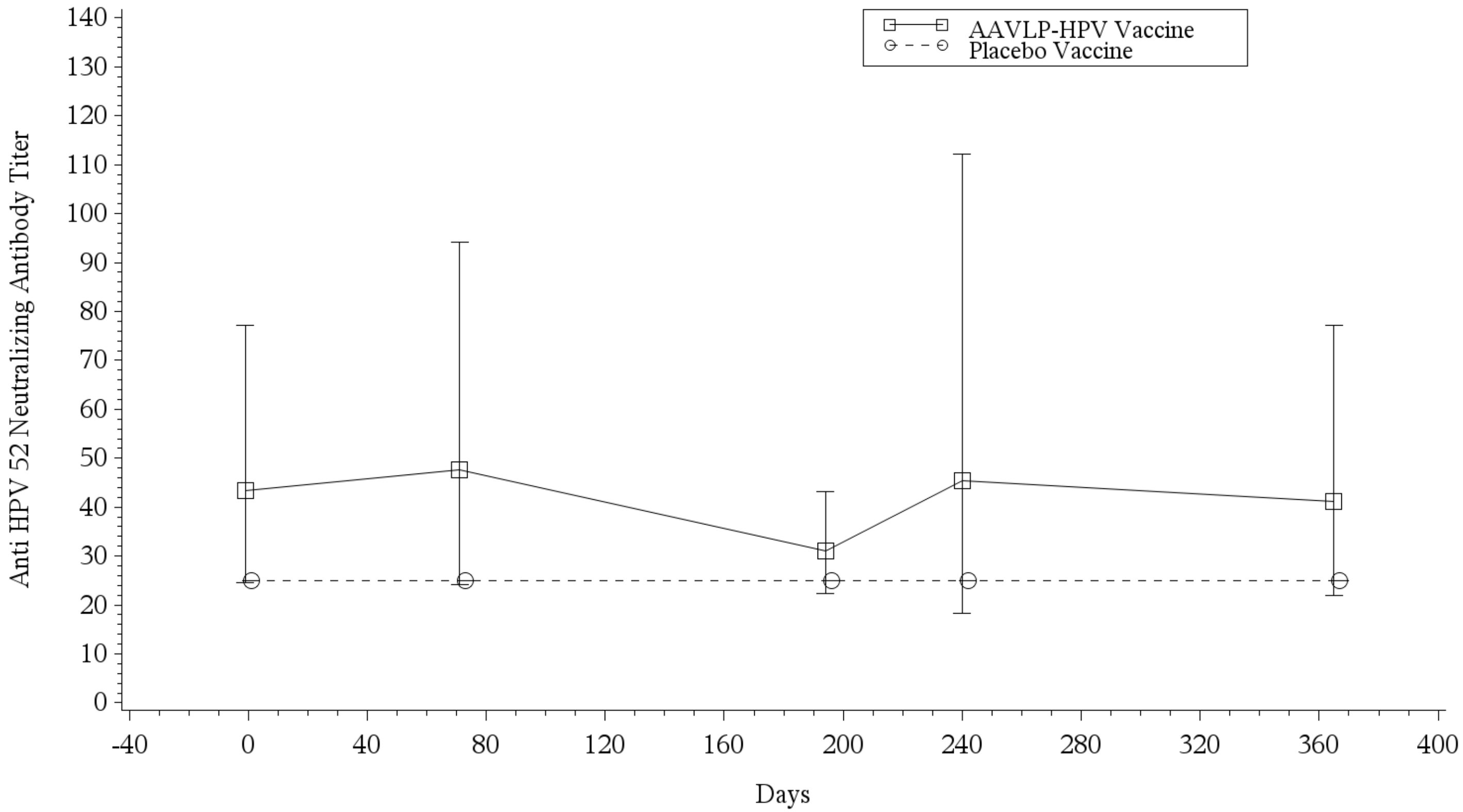

Placebo Vaccine is shifted to the right for ease of reading.
Program: /CA21491/sas_prg/pksas/ada/meangraph.sas 12SEP2022 8:04

Supplementary Figure 13. Geometric Mean (95% CI) Anti HPV58 Neutralizing Antibody Titer Versus Time Profiles Following 3 Intramuscular Injections of AAVLP-HPV Vaccine 20 μg or Placebo Vaccine on Days 1, 57, and 180.

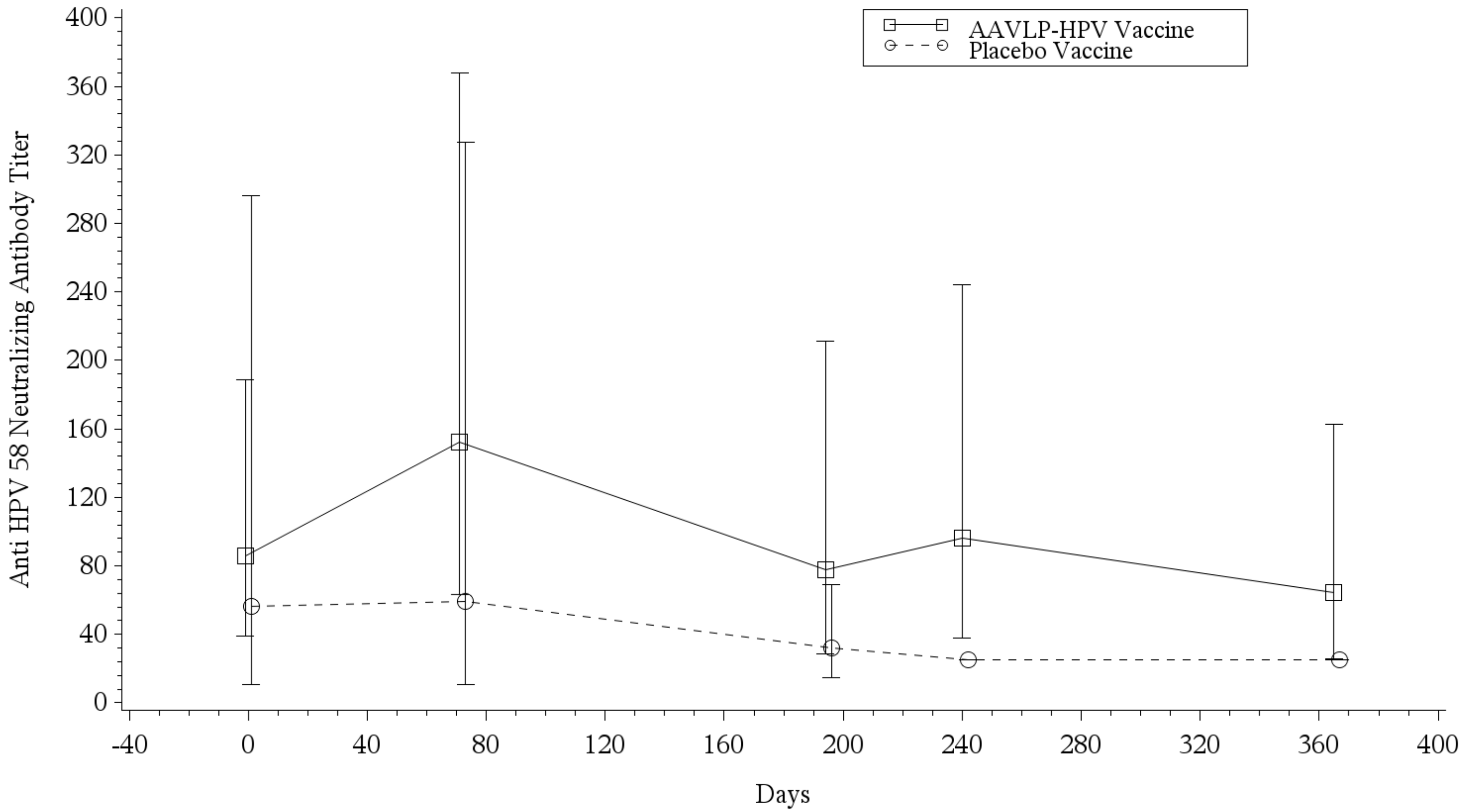

Placebo Vaccine is shifted to the right for ease of reading.
Program: /CA21491/sas_prg/pksas/ada/meangraph.sas 12SEP2022 8:04

Supplementary Figure 14. Geometric Mean (95% CI) Anti HPV59 Neutralizing Antibody Titer Versus Time Profiles Following 3 Intramuscular Injections of AAVLP-HPV Vaccine 20 µg or Placebo Vaccine on Days 1, 57, and 180.

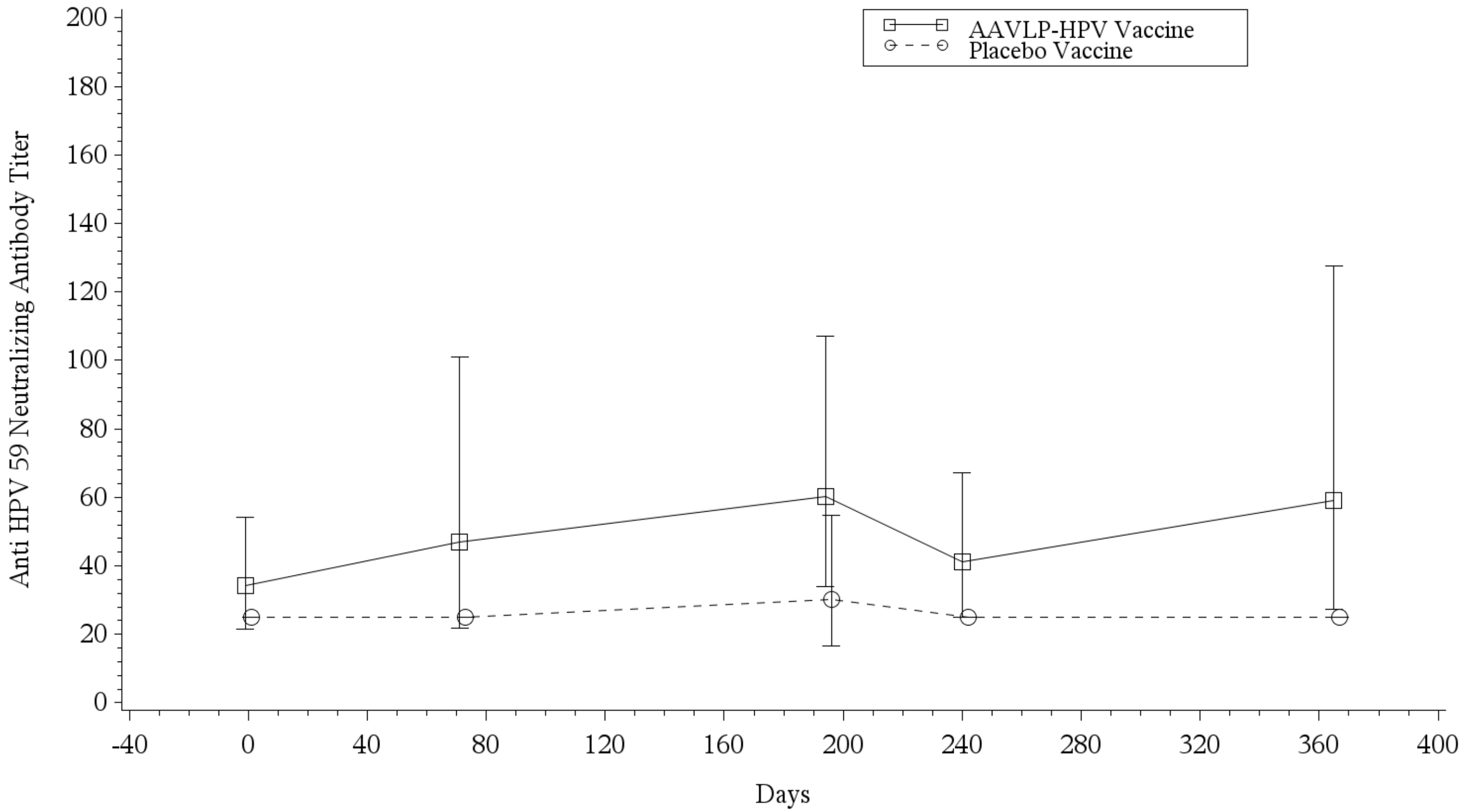

Placebo Vaccine is shifted to the right for ease of reading.
Program: /CA21491/sas_prg/pksas/ada/meangraph.sas 12SEP2022 8:04

Supplementary Figure 15. Geometric Mean (95% CI) Anti HPV68 Neutralizing Antibody Titer Versus Time Profiles Following 3 Intramuscular Injections of AAVLP-HPV Vaccine 20 µg or Placebo Vaccine on Days 1, 57, and 180.

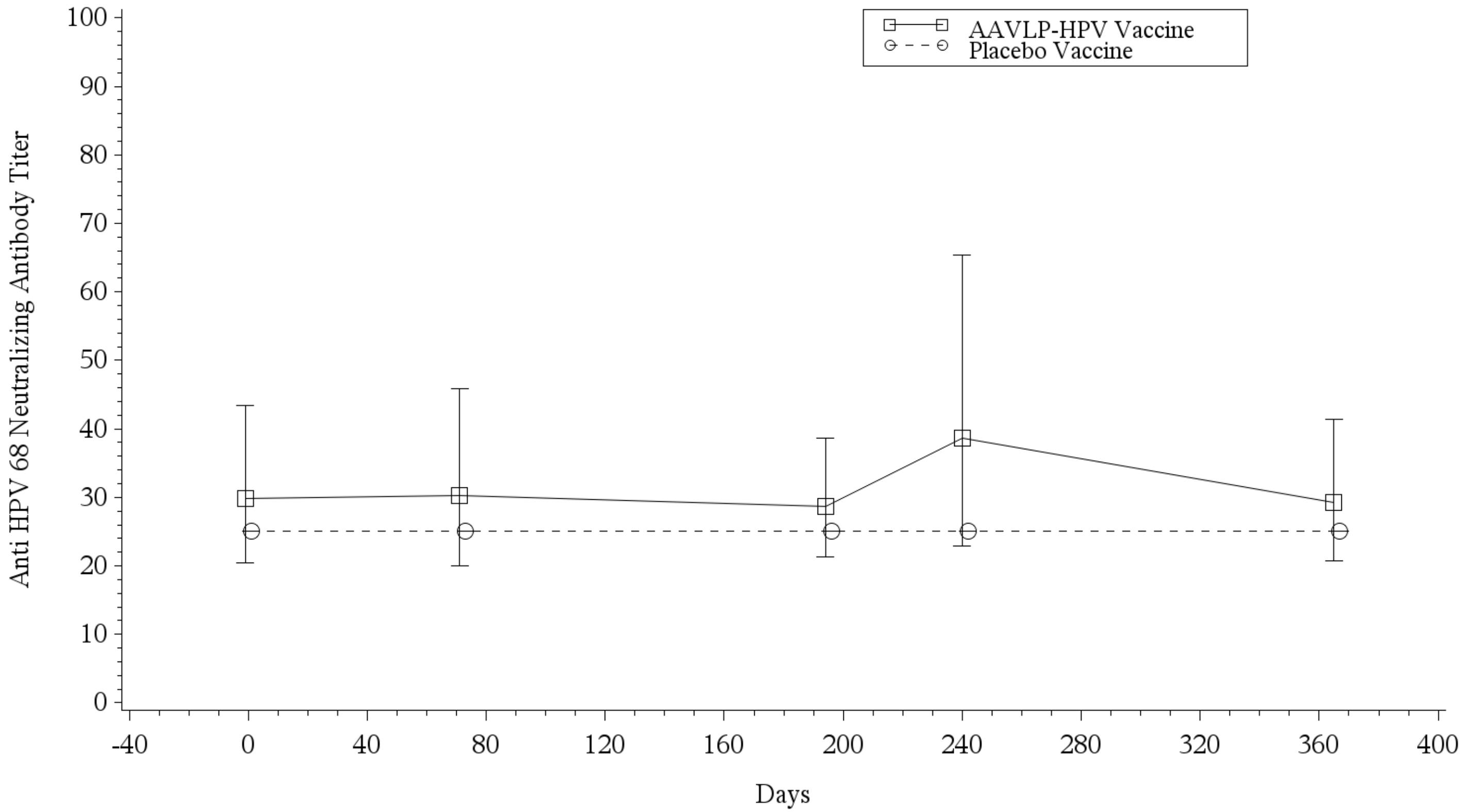

Placebo Vaccine is shifted to the right for ease of reading.
Program: /CA21491/sas_prg/pksas/ada/meangraph.sas 12SEP2022 8:04

Supplementary Figure 16. Geometric Mean (95% CI) Anti HPV73 Neutralizing Antibody Titer Versus Time Profiles Following 3 Intramuscular Injections of AAVLP-HPV Vaccine 20 µg or Placebo Vaccine on Days 1, 57, and 180.

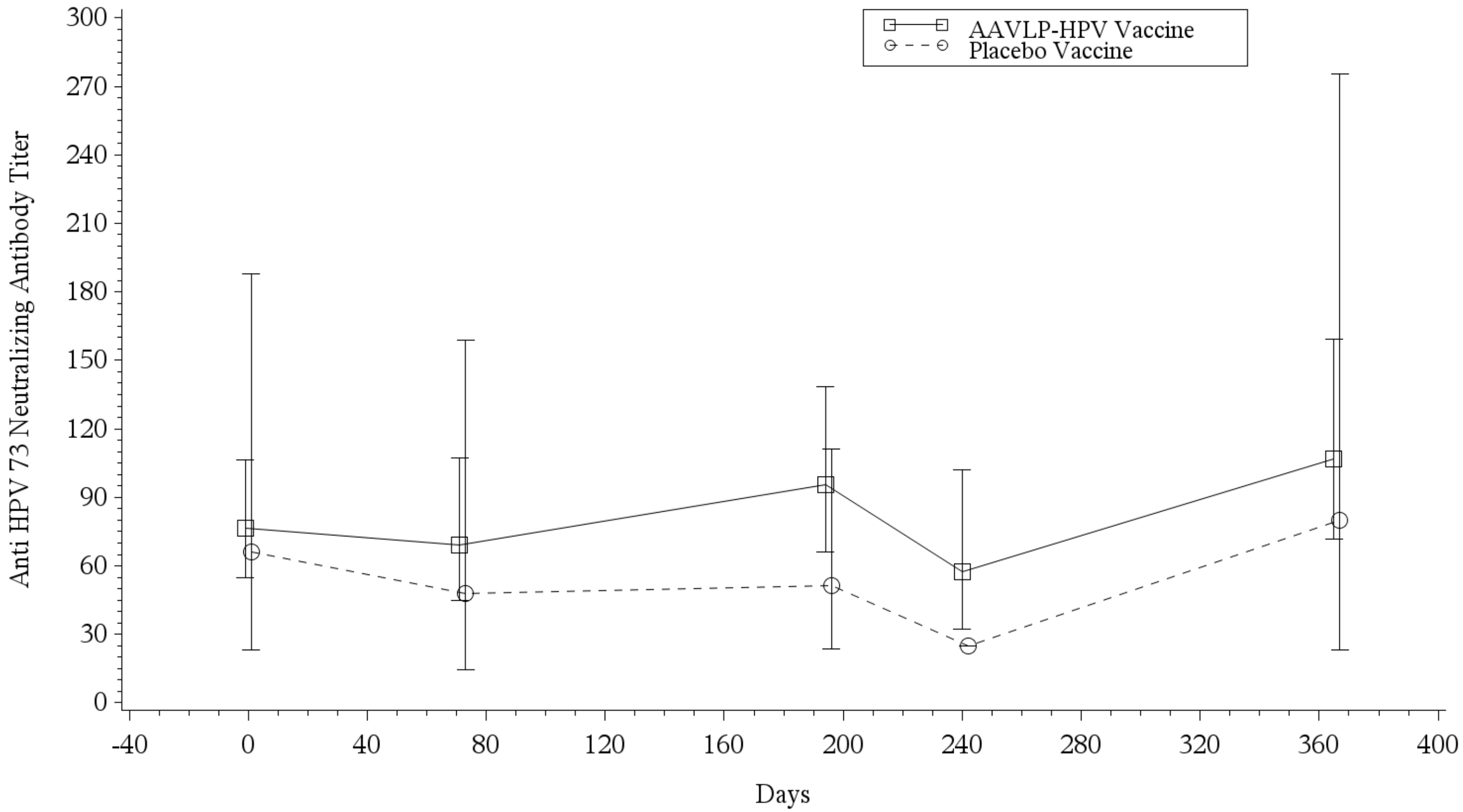

Placebo Vaccine is shifted to the right for ease of reading.
Program: /CA21491/sas_prg/pksas/ada/meangraph.sas 12SEP2022 8:04

Supplementary Figure 17. Geometric Mean (95% CI) Anti HPV92 Neutralizing Antibody Titer Versus Time Profiles Following 3 Intramuscular Injections of AAVLP-HPV Vaccine 20 µg or Placebo Vaccine on Days 1, 57, and 180.

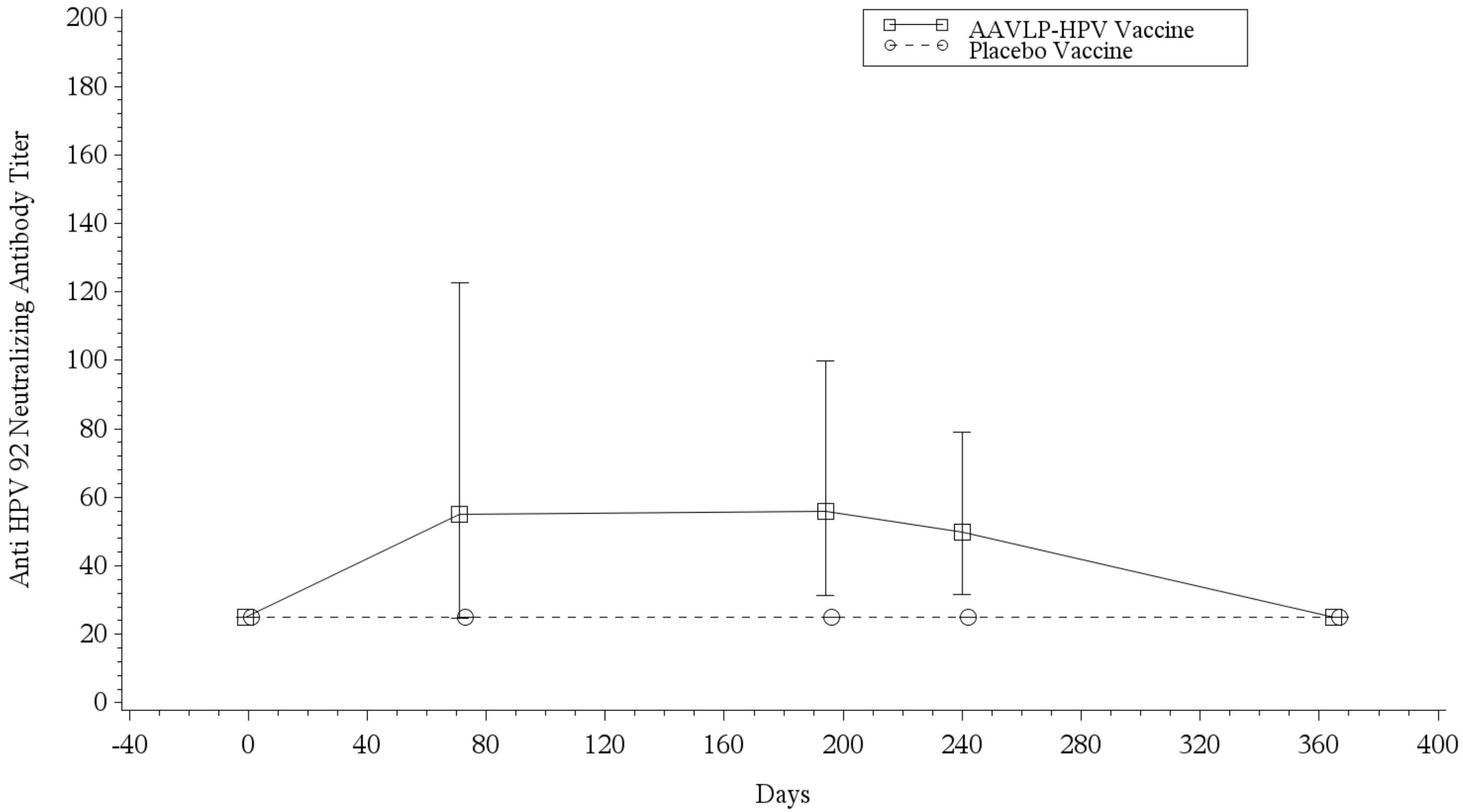

Placebo Vaccine is shifted to the right for ease of reading.
Program: /CA21491/sas_prg/pksas/ada/meangraph.sas 12SEP2022 8:04

111
